# Developmental Potential of Vitrified Mouse Testicular Tissue after Ectopic Transplantation 

**DOI:** 10.22074/cellj.2016.3989

**Published:** 2016-04-04

**Authors:** Nazila Yamini, Gholamreza Pourmand, Fardin Amidi, Mojdeh Salehnia, Nahid Ataei Nejad, Seyed Mohammad Mougahi

**Affiliations:** 1 Department of Anatomy, Faculty of Medicine, Tehran University of Medical Sciences, Tehran, Iran; 2 Urology Research Center, Sina Hospital, Tehran University of Medical Sciences, Tehran, Iran; 3 Department of Anatomy, Faculty of Medicine, Tarbiat Modares University, Tehran, Iran; 4 Department of Histology, Faculty of Medicine, Tehran University of Medical Sciences, Tehran, Iran

**Keywords:** Vitrification, Cryopreservation, Transplantation, Spermatogenesis, Testicular Tissue

## Abstract

**Objective:**

Cryopreservation of immature testicular tissue should be considered as an important factor for fertility preservation in young boys with cancer. The objective of this study is to investigate whether immature testicular tissue of mice can be successfully cryopreserved using a simple vitrification procedure to maintain testicular cell viability, proliferation, and differentiation capacity.

**Materials and Methods:**

In this experimental study, immature mice testicular tissue fragments (0.5-1 mm²) were vitrified-warmed in order to assess the effect of vitrification on testicular tissue cell viability. Trypan blue staining was used to evaluate developmental capacity. Vitrified tissue (n=42) and fresh (control, n=42) were ectopically transplanted into the same strain of mature mice (n=14) with normal immunity. After 4 weeks, the graft recovery rate was determined. Hematoxylin and eosin (H&E) staining was used to evaluate germ cell differentiation, immunohistochemistry staining by proliferating cell nuclear antigen (PCNA) antibody, and terminal deoxynucleotidyl transferase (TdT) dUTP Nick- End Labeling (TUNEL) assay for proliferation and apoptosis frequency.

**Results:**

Vitrification did not affect the percentage of cell viability. Vascular anastomoses was seen at the graft site. The recovery rate of the vitrified graft did not significantly differ with the fresh graft. In the vitrified graft, germ cell differentiation developed up to the secondary spermatocyte, which was similar to fresh tissue. Proliferation and apoptosis in the vitrified tissue was comparable to the fresh graft.

**Conclusion:**

Vitrification resulted in a success rates similar to fresh tissue (control) in maintaining testicular cell viability and tissue function. These data provided further evidence that vitrification could be considered an alternative for cryopreservation of immature testicular tissue.

## Introduction

Cryopreservation of testicular tissue and its potential applications in experimental and clinical settings is a topic of urgency in reproductive medicine (1). Substantial progress has been made in treatment of childhood cancers that have led to an increase in survival rate of approximately 80% (2,4). Unfortunately, gonadal disorders as an adverse effect of gonadotoxic treatments can lead to infertility in approximately one-third of survivors (5). Therefore, preservation of gonadal function will considerably impact the future quality of life of these survivors and should be given due consideration (6,7). 

Since spermatozoa are not produced before puberty, cryopreservation of immature testicular tissue that contain spermatogonial stem cells (SSCs) followed by transplantation or *in vitro* maturation is the only strategy for fertility preservation (8,9), which involves freezing cells and tissues (10). Although cryopreservation of isolated testis cells has been successfully achieved, only in the last 10 years has testicular tissue cryopreservation been investigated. Testicular tissue cryopreservation is still in the experimental stage (7,11). 

Slow freezing (SF) is the conventional method used in most experiments. A few studies have been published that used vitrification. Vitrification is simple, convenient and a more effective method to minimize cellular damage by the use of high concentrations of cryoprotectants in addition to an ultrafast cooling rate which can prevent ice crystal formation (12). Previously, vitrification has been studied to evaluate the efficiency of this strategy in preservation of testicular tissue. Complete spermatogenesis development was obtained after xenotransplantation of vitrified porcine testicular tissue into nude mice (11). Testicular tissue vitrification in non-human primates followed by xenotransplantation into nude mice showed maintenance of functional Leydig cells, integrity preservation of testicular tissue, and proliferating spermatogonia (13). In mice, vitrification resulted in normal preservation of seminiferous tubule integrity and proliferating activity after 3 days of organotypic culture (14). Although apoptosis might be induced by slow cooling, evidence has shown that high concentrations of cryoprotectants used in vitrification may induce apoptotic pathways (14,15). 

Despite a number of vitrification protocols that have been studied in different species, a distinctly established procedure does not exist (16,17). Continued efforts directed toward improvement of cryopreservation protocols are necessary for optimization of post-thaw testicular cell functionality (15). An important factor in evaluating efficiency of a cryopreservation protocol for a multicellular structure, such as gonadal tissue, is the evaluation of tissue functionality in addition to an assessment of post-thaw cell viability rates. Achieving high cellular viability does not always result in preservation of the tissue’s developmental potential (11,12). Therefore, transplantation of the cryopreserved tissue allows a longer-term functional assessment both in terms of cell proliferation and germ cell differentiation (11,17). 

To the best of our knowledge, no study has evaluated the developmental potential of vitrified mice testicular tissue *in vivo*. The objective of this study was to test the efficiency of a simple vitrification and warming procedure to preserve the functional capacity of immature mice testicular tissue that was ectopically transplanted into castrated mice with normal immunity. 

## Materials and Methods

### Animals

This experimental study used 6-day-old postnatal male BALB/c mice as the testes donors and 8-10-week-old males of the same strain (n=7 for vitrified testicular tissue and n=7 for fresh) as testes recipients. Mice were acquired from Pasture Institute of Iran. Animals were kept and bred in the colony room with access to water and chow. 

Animals were maintained under controlled conditions (12-hour light: 12-hour dark). The experiments were carried out in accordance with the Tehran University Guideline for the Care and Use of Laboratory Animals. 

### Preparation of immature testicular tissue

Donor intra-abdominal testes were surgically removed and immediately transferred to Dulbecco’s modified Eagle’s medium (DMEM, USA, Gibco) supplemented with 10% fetal bovine serum (FBS, USA) on ice. The tunica albuginea was removed and the testes were fragmented in 0.5-1 mm² pieces. We chose this small size in order to enhance tissue vascularization and survival (18). These tissue pieces were randomly divided into 3 groups: control (fresh non-vitrified, n=47), vitrified (n=47), and solution control (n=4). 

### Vitrification and warming

We selected the vitrification and warming protocols according to promising results from a previous study (11,13,19). For vitrification, approximately 2-3 testicular tissue fragments were put into an equilibration solution that consisted of 7.5% (v/v) dimethylsulfoxide (DMSO, Sigma, USA) 7.5% (v/v) ethylene glycol (EG, Sigma, USA), 0.25 M sucrose (Sigma, USA) and 10% FBS in DMEM-F12 (Gibco, USA) for 10 minutes at 4˚C. The fragments were transferred to a vitrification solution that consisted of 15% DMSO, 15% EG, 0.5 M sucrose and 10% FBS in DMEM-F12 for 5 minutes at 4˚C. To achieve maximum cooling rate with minimum the vitrification medium around the tissue. Immediately, we used very fine forceps to place 2-3 fragments on a metal grid (20) which was plunged into liquid nitrogen (LN_2_) and inserted into precooled cryotubes. At one week after cryopreservation the samples were thawed. For warming, the cryotubes were removed from LN_2_and the metal grid were quickly immersed in a 35˚C warming solution that contained sucrose (1 M) in DMEM-F12 with 10% FBS for 1 minute, after which they were transferred to 3 baths of warming solutions of decreasing sucrose concentrations (0.5, 0.25, and 0 M) for 3 minutes each. 

### Solution test

In order to investigate the toxicity effects of the vitrification and thawing solutions on the testicular tissue, the fragments were passed through all stages of the vitrification and thawing steps without plunging in LN_2_. 

### Assessment of cell viability

We used the Trypan blue test to determine cell viability after enzymatic digestion of tissue fragments to a single cell suspension. Enzymatic digestion was performed on the fresh, toxicity test and vitrified-warmed testis fragments. The testis pieces were exposed in DMEM that contained 0.2% wt/vol collagenase type IV (Sigma-Aldrich, USA) at 37˚C for 8 to 10 minutes with occasional agitation, followed by the addition of 0.01% wt/ vol DNase type I (Sigma, USA) in DMEM for an additional 5 to 10 minutes. The sample was centrifuged at 500 ×g and the supernatant was removed. We calculated cell viability by adding an equal volume of Trypan blue (0.4% solution, Sigma, USA) to an equal volume of the cell suspension. The solution was allowed to incubate at room temperature for approximately 3 minutes. The sample was placed on a hemocytometer and observed under a bright-field microscope at ×400 magnification. A total of 200 cells were counted. Clear cells were considered to be viable since Trypan blue cannot penetrate through a healthy cell membrane. Blue cells were considered nonviable. Viability was calculated as follows:

% viable=(number viable cellsnumber total cells×100)

### Transplantation of testicular tissue fragments

The recipient mice were anesthetized by an intraperitoneal injection of a mixture of 80 mg/ kg ketamine and 10 mg/kg of xylazine (Upjohn, Germany). After surgical preparation the recipient mice, during the same surgery, underwent castration just prior to testicular grafting through scrotal incisions (20). Six pieces of fresh or vitrified donor testes (0.5-1 mm²) were ectopically grafted to each host under the back skin on either side of the dorsal midline using a syringe needle plunger system as described by Ma et al. (21). Briefly, a fragment of the testicular tissue (0.5-1 mm) was inserted into the tubing of a 16-gauge needle [inner diameter (ID) 1.0 mm, outer diameter (OD) 1.2 mm], which penetrated to the dorsal skin of the mice. The testis was placed under the skin by pushing a fine steel wire inside the tubing of the needle. 

### Assessment and collection of grafting

The recipient mice were killed after 5 weeks by cervical dislocation. The site of transplantation was carefully dissected. The number of detectable grafts were recorded and removed, then fixed in Bouin’s solution. 

After fixation and routine histological processing, samples were embedded in paraffin. We prepared 3 (5 µm thick) sections from the largest diameter of each sample at intervals of 20 µm and sustained with hematoxylin and eosin (H&E). The slides were coded and observed under a light microscope equipped with a digital camera (Olympus AX70, Japan) to evaluate the degree of spermatogenesis activity and the most uadvanced stage of germ cell development. All seminiferous tubules present in each histological section were classified as either Sertoli cell only tubules or tubules with the most advanced germ cells. The percentages of seminiferous tubules that showed differentiation were determined. The total numbers of intratubular cells (spermatogonia, Sertoli cells and spermatocytes) were also counted in four microscopic fields ×40 (50 µm) selected randomly in each of the grafted sections. 

### Immunohistochemistry

In order to evaluate proliferating cells, tissue blocks were sectioned into 5 µm sections. Briefly, sections were dewaxed in xylene (Merck, Germany) followed by rehydration in decreasing grades of ethanol. Sections were permeabilized with 0.2% Triton X-100 (Sigma, USA) and non-specific binding sites were blocked using 1% normal horse serum for 30 minutes. Primary antibody against proliferating cell nuclear antigen (PCNA, Abcam, USA) was added to the sections, followed by an overnight incubation at 4˚C. To probe primary antibody binding sites, biotinylated universal secondary antibody (VECTASTAIN Universal Elite ABC Kit, Vector Laboratories, Burlingame, USA) was incubated for 30 minutes. Using Diaminobenzidine (DAB) chromogen (Vector Laboratories, Burlingame, USA) positive cells were revealed. Counterstaining was performed by hematoxylin, after which cells were mounted with Entellan (Merck, Germany). Slides were observed by a light microscope (BX41, Olympus, Japan) and the mean number of positive cells in five microscopic fields (×40) was calculated. 

### Terminal deoxynucleotidyl transferase dUTP Nick-End Labeling assay

Apoptotic cells in sections were detected by the TUNEL assay. Sections were probed using a Roche kit according to the manufacturer’s instructions. Briefly, sections were dewaxed, dehydrated and permeabilized by 15 µg/ml proteinase K for 20 minutes at 37˚C (Roche, Germany). TUNEL reaction mixture was added to sections. The sections were allowed to incubate for 1 hour at 37˚C. After several PBS washes, sections were incubated with Converter-POD for 30 minutes at 37˚C. DAB, as a chromogenic substrate of horse radish peroxidase (HRP), was applied to distinguish TUNEL positive cells. Counterstaining was performed by hematoxylin, then cells were mounted with Entellan (Merck, Germany). Slides were observed by a light microscope (BX41, Olympus, Japan) and the mean number of positive cells in five microscopic fields (×40) were calculated. 

### Statistical analysis

Data were presented as the mean ± standard error. ANOVA and the student’s t test were utilized to compare data using the Statistical Package for the Social Sciences, Version 18.0 software (SPSS Inc., USA). P values less than 0.05 were considered statistically significant. 

## Results

### Cell viability

**Fig.1:**
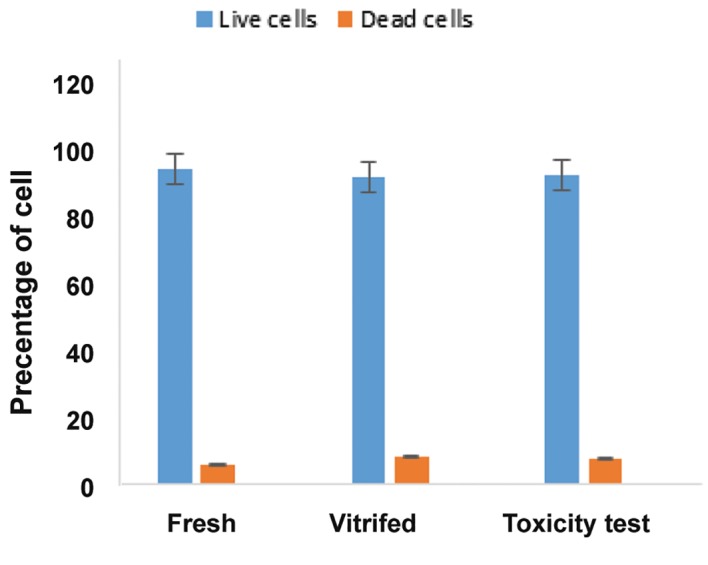
Viability of fresh, vitrified and toxicity test by Trypan blue staining, No statistically significant difference in cell viability was observed between vitrified, solution test, and fresh fragments.

Testicular cell viability was 94.2 ± 1.158 (fresh control), 92 ± 1.095 (vitrified), and 92.4 ± 0.927 (solution test).Viability in the vitrified and solution test were comparable with the control. We observed no significant difference in cell survival rate between the samples (Fig.1).

### Survival of the testis graft

**Fig.2:**
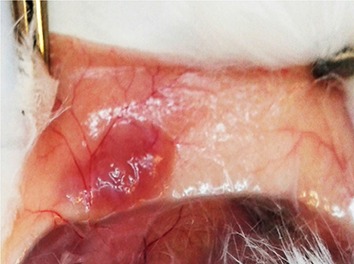
Representative micrograph of subcutaneous site of testis graft in host mice 4 weeks after grafting. Note the presence of the revascularization surrounding the transplant location to provide enough blood to the graft.

**Fig.3:**
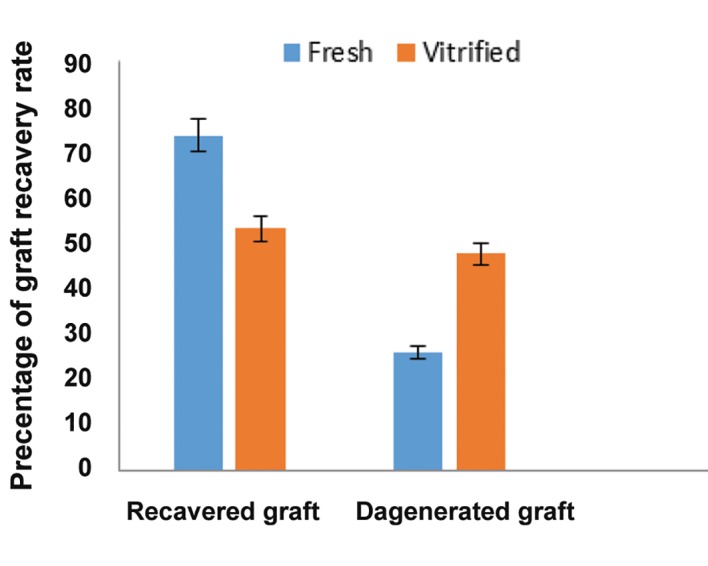
Graft recovery rate. At 4 weeks post-transplantation, the percentage of recovered graft showed no significant difference in the rate of surviving graft harvested from fresh (control) and vitrified-warmed tissue.

The survival and growth of the grafted tissue was easily observed under the back skin of the recipient mice. Figure 2 shows a typical example of the back skin with surviving vitrified graft. At 4 weeks after transplantation, 73.8% (32/42) of the fresh grafts and 52.3% (22/42) of the vitrified grafts were recovered. Graft recovery is defined as the detectable graft collected in the weeks after transplantation. This difference was not statistically significant between the fresh and vitrified groups (Fig.3). 

### Evaluation of testicular differentiation

**Fig.4:**
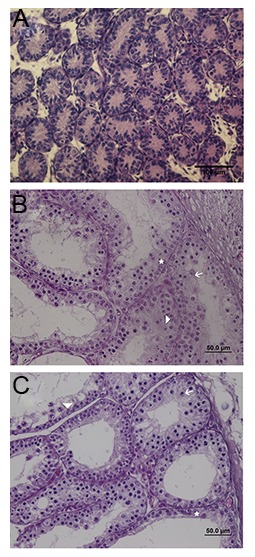
Histologic appearance of hematoxylin and eosin (H&E) fresh and vitrified immature mice testis tissue before and after grafting. A. Fresh immature mice testis tissue at the time of grafting, B. Testis tissue recovered at 4 weeks post-graft from fresh (control) and C. Vitrified. Arrow; Spermatocyte, Arrow head; Sertoli cell and Asterisks; Spermatogonia (scale bar: 50 μm at ×40 magnification).

**Fig.5:**
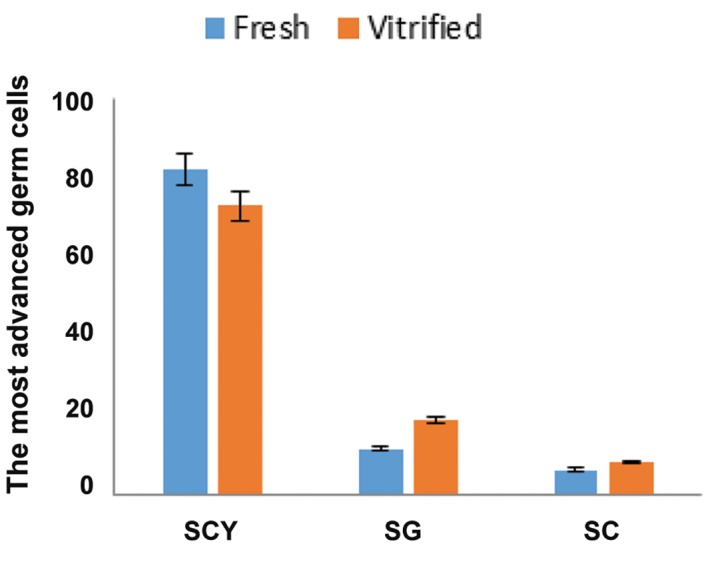
The most advanced germ cell types present in the seminiferous tubules of recovered testis tissue fragments at 4 weeks post-graft. Fresh (control) and vitrified groups showed no statistically significant differences. SCY; Spermatocyte, SG; Spermatogonia and SC; Sertoli cell.

**Fig.6:**
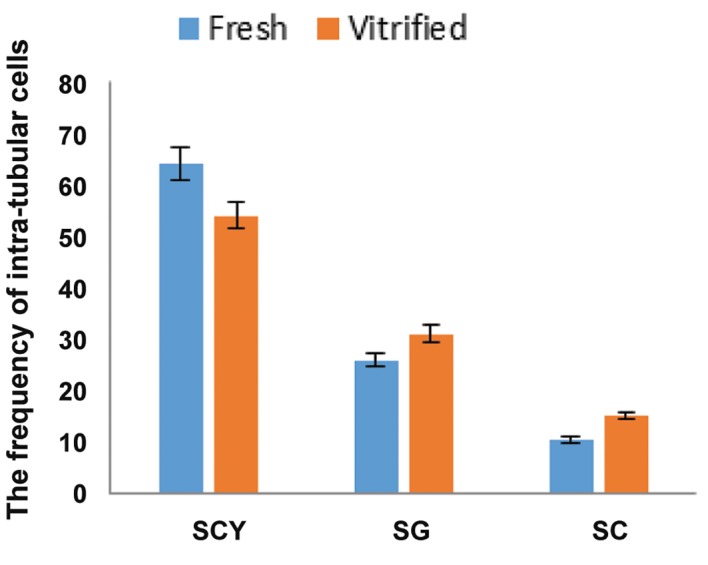
The frequency of intra-tubular cells of the most advanced germ cell types present in the seminiferous tubules of recovered testis tissue fragments at 4 weeks post-grafting. There was no statistically significant difference in the frequency of intra-tubular cells between the fresh and vitrified groups. SCY; Spermatocyte, SG; Spermatogonia and SC; Sertoli cell.

Prior to grafting, the somatic Sertoli cells and gonocytes/ spermatogonia were the only cells present in the seminiferous tubules of neonatal donor tests (Fig.4A). Four weeks after grafting, the seminiferous tubules of fresh (control) and vitrified graft harvested showed variable degrees of spermatogenic germ cells (Fig.4B, C). The percentage of the most advanced germ cells present in the tubules of fresh (control) and vitrified graft recovered respectively was calculated as follow: Sertoli cell only (6.25% ± 0.6023 and 8.20% ± 1.844), spermatocyte (82.2% ± 1.504 and 73% ± 3.827) and spermatogonia (11.50% ± 1.157 and 18.80% ± 2.156) (Fig.5), and also the frequency of intratubular cell per microscopic field (50 μm=×40) was respectively included in spermatocyte(64% ± 1.197 and 54% ± 1.898), spermatogonia (25.70% ± 2.512 and 31% ± 4.013)and Sertoli cell (10.30% ± 1.959 and 15% ± 2.487) in fresh and vitrified graft (Fig.6). The potential for spermatogenesis development in the vitrified graft was similar to the fresh. We observed no statistically significant difference in the most advanced germ cells and frequency of intratubular cells between the fresh and vitrified groups.

### Apoptosis and cell proliferation ability

**Fig.7:**
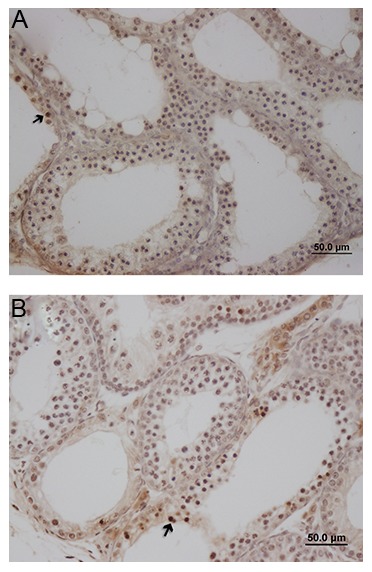
The frequency of TUNEL positive cells 4 weeks post-transplantation. TUNEL positive cells are shown by their deep brown color in A. Fresh and B. Vitrified tissues (scale bar: 50 μm at ×40 magnification). Arrow; Positive cell.

Sections stained by TUNEL were analyzed to evaluate for the presence of apoptosis after vitrification. The frequency of apoptotic cells per microscopic field did not differ from the fresh (control). The number of apoptotic cells slightly increased after vitrification in the fresh (4.5 ± 0.93) and vitrified (7.5 ± 1.16) groups, but did not reach statistical significance (Fig.7).

**Fig.8:**
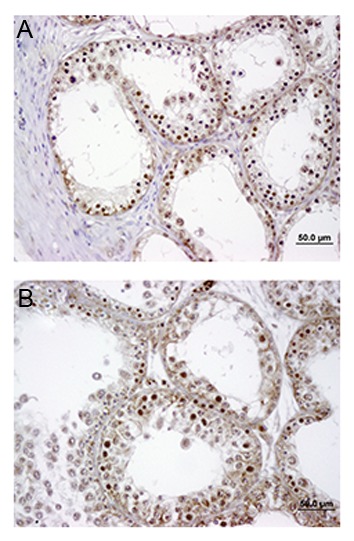
The frequency of proliferating cell nuclear antigen (PCNA) positive cells 4 weeks post-transplantation. PCNA positive cells are shown by the deep brown color in A. Fresh and B. Vitrified tissues (scale bar: 50 μm at ×40 magnification).

After grafting of vitrified tissue, we observed slightly decreased cell proliferation in the vitrified group (14.6 ± 2.16) compared with the fresh (control) group (21.3 ± 2.8), however this finding was not statistically significant (Fig.8).

## Discussion

Cryopreservation of testicular tissue can open new possibilities for preservation of reproductive activity. Cryopreservation is useful in practical and clinical applications including preservation of the germ lines of valuable and immature threatened species and conservation of fertility in pre-pubertal boys who undergo cancer treatment (11). Vitrification is a fast, convenient and efficient technique widely used for oocytes and embryos. This technique also has promising results in preservation of ovarian tissue in several species (12). Recently, numerous attempts have been made to investigate the efficiency of maintaining testicular tissue. In the present study, we have examined whether immature testicular tissue of a mice species could be successfully cryopreserved using a simple verification procedure in order to maintain high numbers of viable testicular cells as well as proliferation and differentiation capacity.

There are three critical factors to achieve a successful vitrification which include a high cryoprotectant concentration, small volume, and high cooling as well as warming rate (22). At present, DMSO as a cryoprotectant has shown the most promising results to preserve immature testicular tissue in animals and humans. Because the toxicity of high DMSO concentrations is well-known (23-25), a combination of EG and DMSO has been suggested to obtain a less toxic vitrification solution (25). However, this efficiency differs between species. We have based the concentrations of EG and DMSO in the present study on those used for the successful vitrification of porcine tissue (11). According to the literature, the highest rate of post-cryopreservation viability by avoiding crystallization has resulted in a high survival rate (26). One way to achieve a higher cooling rate has been facilitated by the use of a special carrier such as cryoloops , cryotops and needles (27, 28). In this regard, several studies have evaluated different carrier systems during the vitrification process, including open pulled straws (OPS) (13, 29). Solid surface vitrification (SSV) has been used to successfully vitrify immature piglet testicular tissue (11). However there was a negative effect on the number of human spermatogonia, this would be related to an inadequate cooling rate as result of which crystallization and cell damage would appear (23). In this study, based on encouraging results obtained by ovarian tissue vitrification, we used a metal grid described by Kagawa et al. (19), which was modified to a smaller size. In order to evaluate a vitrification procedure in addition to cell viability and tissue integrity, the functional assessment test is also important (11, 24).

The results of the first part of our study demonstrated that the cryoprotectant composition and cooling and warming rates used in this experiment did not affect the percentage of cell viability. Also our functional assessment by transplantation resulted in successful revascularization in the graft site in addition to the number of functional grafts harvested that had germ cell proliferation and differentiation up to secondary spermatocyte in the vitrified graft. Our result was compatible with that of the fresh recovered graft. This finding agreed with results in piglet vitrified tissue, which achieved high cell viability after vitrification in addition to successful spermatogenesis development following xenotransplantation (11, 30). On the other hand, promising results obtained from the first part of human testicular vitrification with well-preserved seminiferous tubule structural integrity post-cryopreservation did not guarantee in maintenance of tissue developmental potential 6 months after transplantation (25), then the vitrification protocol should be confirmed by the efficiency of which at each level of evaluation.

## Conclusion

We evaluated the efficiency of mice testicular tissue vitrification after ectopic transplantation. The results showed success rates similar to fresh (control) tissue in maintaining testicular cell viability and tissue function. These data provide further confirmation that vitrification could be suitable for cryopreservation of immature testicular tissue. However, longer grafting periods would be necessary in order to demonstrate the capacity of spermatogenesis to expand beyond to spermatocytes.
